# Immune risk phenotype and long-term mortality after community-acquired pneumonia in older adults: clinical and immune determinants

**DOI:** 10.1186/s12979-026-00574-8

**Published:** 2026-06-02

**Authors:** Sandra Clotet-Vidal, Teresa Franco-Leyva, Encarna Saez Prieto, Laura Feltrer Martinez, Alvaro Izquierdo Cardenas, Laura Martínez-Martínez, Jordi Casademont Pou, Olga H. Torres Bonafonte

**Affiliations:** 1 Internal Medicine Department, Hospital de la Santa Creu i Sant Pau, Sant Antoni M. Claret 167, Barcelona, 08025 Spain; 2 Immunology Department, Hospital de la Santa Creu i Sant Pau, Sant Antoni M. Claret 167, Barcelona, 08025 Spain; 3 Geriatrics Unit, Internal Medicine Department, Hospital de la Santa Creu i Sant Pau, Sant Antoni M. Claret 167, Barcelona, 08025 Spain; 4 Medicine Department, Universitat Autònoma de Barcelona, Barcelona, 08913 Spain; 5 Institut de Recerca Sant Pau (IR SANT PAU), Sant Quintí 77-79, Barcelona, 08041 Spain

**Keywords:** Immunosenescence, Older adults, Community-acquired pneumonia, Prognosis

## Abstract

**Background:**

Community-acquired pneumonia (CAP) has been associated with poor long-term outcomes in older adults. In most pre-pandemic studies, long-term mortality was assessed with a focus on comorbidity burden. Although immunosenescence is a key determinant of CAP, post-acute immune patterns have been little explored.

**Objective:**

To assess 18-month mortality after CAP hospitalisation and the prognostic value of the Immune Risk Phenotype (IRP).

**Methods:**

Prospective, observational study of adults ≥ age 65 years discharged after CAP (2019—21). We performed comprehensive geriatric and nutritional assessments, including laboratory tests at 30–60 days post diagnosis. IRP was defined as cytomegalovirus seropositivity with inverted CD4:CD8 ratio, elevated CD8 + T cell count, or expansion of CD8CD28-T cells. The main outcome measure was 18-month mortality. Multivariate logistic with ROC curves and Cox regression analyses were performed. (ClinicalTrials.gov, NCT0462799).

**Results:**

The sample included 143 patients (55.2% males), with a mean age of 77.6 ± 7.9 years. IRP was found in 46.8% of patients. Elevated lymphocyte count and female sex were predictors of IRP. At 18 months post-CAP, 27 patients (18%) had died, mainly due to infections (*n* = 13, 48%) and fractures (*n* = 2, 7.4%). Predictors of 18-month mortality included IRP (HR 2.58 [95% CI 1.11–5.99]; *p* = 0.027), severe chronic kidney disease (6.16 [2.53–14.99]; *p*<0.001), poor functional status (3.69 [1.53–8.90] *p* = 0.004), and hypoalbuminemia (3.35; [1.34–8.41]; *p* = 0.010).

**Conclusions:**

This study underscores the role of infectious complications in long-term outcomes after CAP. These findings highlight the need for comprehensive geriatric assessment to identify multidimensional risk factors, including immunosenescence as a potentially modifiable domain warranting further investigation and integration into care.

**Supplementary Information:**

The online version contains supplementary material available at 10.1186/s12979-026-00574-8.

## Background

Community-acquired pneumonia (CAP) remains a major health concern, particularly among older adults. The estimated annual incidence of CAP in Europe ranges from 1.07 to 1.2 cases per 1,000 people, but the incidence is substantially higher in people over age 65 (14 cases per 1,000) [1]. CAP is a common cause of hospitalisation in this older population and associated with high short-term morbidity and mortality [1, 2]. Furthermore, previous studies have reported one-year mortality rates ranging from 12% to 17% in older adults following hospitalisation for CAP [3–7].

Studies also demonstrated higher long-term mortality rates in these patients compared to those hospitalised for other medical conditions [6, 8]. Key predictors of poor long-term prognosis include comorbid conditions, social factors (e.g., nursing home residence), and poor functional status [7–9]. Although it is evident that CAP places a significant long-term burden on patients, the mechanisms underlying the increased mortality remain poorly understood [6].

One of the many factors thought to contribute to the increased susceptibility to infections and the decreased efficacy of vaccination in older adults is age-related changes in the immune system, also known as immunosenescence, which involves both innate and adaptive immunity [10, 11]. Among the characteristic changes of immunosenescence, those considered to play a major role in the age-related decline of the immune system include the progressive decline in naïve circulating T-cell counts, an expansion of memory T-cells, and the accumulation of terminally-differentiated effector CD8 T-cells [11, 12]. In particular, infection with the cytomegalovirus (CMV)—a common herpes virus—is considered to be one of the main repeated antigenic stimuli responsible for the accumulation of oligoclonal effector CD8 T cells [13]. In recent years, researchers have identified a set of immune-related parameters, known as the “immune risk phenotype” (IRP), to characterise patients at high risk of adverse outcomes. These parameters include an inverted CD4/CD8 ratio (< 1), expansion of terminally-differentiated cytotoxic T cells, and CMV seropositivity [12, 13]. IRP has been identified as an indicator of both in-hospital mortality and nosocomial pneumonia [11]. To our knowledge, however, the potential role of IRP as a long-term prognostic factor in older adults hospitalised for an infectious disease such as CAP has not been evaluated to date.

Nutritional status is a major contributor to immune-system development and plays a key role in immune function. This is particularly important in older adults because many have poor nutritional status and insufficient intake of vitamins and minerals [14]. Malnutrition increases susceptibility to infections, and is a risk factor for worse clinical outcomes in CAP patients; moreover, an episode of CAP may further worsen nutritional status due to disease-related malnutrition [15]. Protein energy malnutrition plays a crucial role in ensuring the proper functioning of the immune system [16]. Other nutritional deficits, including micronutrient deficiencies, may also influence immune responses, although their clinical relevance remains uncertain [14]. Nonetheless, it seems plausible that malnutrition could be involved in immunosenescence.

To date, only limited research has been performed to evaluate immunosenescence or nutritional status as possible risk factors for poor long-term prognosis after an episode of CAP in older adults. In this context, the main aims of this prospective study were (1) to evaluate 18-month mortality after hospitalisation for CAP in a series of older adults and (2) to assess the prognostic value of IRP and to determine its association with clinical markers, particularly nutrition-related markers.

## Methods

### Study design

This was a prospective cohort study conducted at the Hospital de la Santa Creu i Sant Pau, a tertiary university hospital in Barcelona, Spain. All patients admitted to the hospital for pneumonia during the study period who met the inclusion criteria were invited to participate. All participants provided written informed consent.

This study was approved by the hospital ethics committee on 27 February 2019 (approval no.19/068 R-OBS). All study-related procedures were performed in accordance with the 1964 Helsinki Declaration and its later amendments. This prospective, observational, single-centre study aimed to evaluate the prognostic value of multiple biomarkers in older patients admitted for CAP (ClinicalTrials.gov: NCT0462799).

### Participants

Patients aged 65 or older with radiologically-confirmed pneumonia requiring hospitalisation between May 2019 and July 2021 were invited to participate.

Pneumonia was defined as the presence of a new pulmonary infiltrate on X-ray or computed tomography and at least one of the following symptoms: fever, shivering, cough, expectoration, or malaise. The attending physician was responsible for the initial evaluation of the chest radiograph. However, at study inclusion, the authors (OHTB, SCV, AIC, ASG, LFM) reviewed the X-ray images to confirm the diagnosis. In case of disagreement or uncertainties, experienced radiologists were consulted to make a final determination. The attending physician was responsible for all aspects of patient management.

Exclusion criteria were as follows: hospitalisation for ≥ 72 h within the previous 15 days; HIV infection; severe neutropenia (absolute neutrophil count < 1000 × 10^9^/L); chronic lymphocytic leukaemia; transplant recipient status; and/or end-of-life clinical status.

### Data collection and measures

At admission, we recorded the following: demographic data; previous institutionalisation (e.g., nursing home or long-term care facility); Barthel Index (BI) [17] in the 15 days prior to admission for CAP; and the Pneumonia Severity Index (PSI) [18]. Pathogens in samples obtained from sputum, blood, and/or other body fluids were studied using standard microbiological procedures. All patients included after March 2020 were tested for COVID-19 (SARS-CoV-2) through either a polymerase chain reaction test or rapid antigen test. For all patients included between January 2020 and March 2020, serological testing for COVID-19 was performed (negative in all cases).

To ensure that acute events would not influence long-term outcomes, only those patients who survived ≥ 30 days after diagnosis were included in the study. Between 30 and 60 days after the CAP episode, participants underwent comprehensive geriatric assessment (CGA) and a general nutritional assessment (GNA) at the geriatric day hospital. A complete blood test, which included immunological and nutritional parameters, was also performed. At that same visit, we also recorded the patients’ institutionalisation history, BI values, and the Charlson Comorbidity Index (CCI) score [19]. The presence of chronic kidney disease (CKD) was determined with the CCI (CKD defined as presenting one of the following: serum creatinine level > 265 pmol/L, renal transplant, or uraemia) and according to the KDIGO guidelines (glomerular filtration rate [GFR] < 30 mL/min per 1.73 m^2^) [20].

Frailty was defined according to Fried’s criteria (≥ 3 of the following variables): weight loss (10 kg in past year); self-reported exhaustion; weakness (grip strength test); slow walking speed; low physical activity [21].

The GNA included determination of the body mass index (BMI) and the revised version of the Mini Nutritional Assessment (MNA, Nestle Nutrition Institute), a risk screening test for malnutrition [22]. The blood test included nutritional parameters (albumin, B group vitamins, folate, zinc, vitamins C and D), immunological parameters (peripheral lymphocyte phenotyping), and CMV serology.

Based on the MNA scores, nutritional status was categorised as follows: adequate (> 24 points); at risk of malnutrition (17 to 23.5); and protein–calorie malnutrition (< 17) [22]. The methods used for micronutrient determinations and laboratory reference values have been detailed elsewhere [23].

### Immune risk phenotype

The presence of IRP was defined as CMV seropositivity with at least one of the following: inverted CD4:CD8 ratio, elevated CD8 + T cell count (> 600/pl), or expansion of CD8CD28-T cells (> 300/pl) [12]. Peripheral venous blood collected in 3 mL EDTA-coated tubes (BD Vacutainer) was stained with two pre-made commercial mixtures to analyse the lymphocyte subpopulations: CD45-FITC/CD3-PC5/CD4-RD1/CD8-ECD and CD45-FITC/CD56-RD1/CD19-ECD/CD3-PC5 (Beckman Coulter; Brea, CA, USA). The following monoclonal antibodies were used to determine the T lymphocyte differentiation phenotype: anti-CD3 (Beckman Coulter), anti-CD4 (Beckman Coulter), anti-CD8 (Beckman Coulter), anti-CD28 (Sysmex, Norderstedt, Germany), and anti-CD27 (BD Pharmigen, Franklin Lakes, NJ, USA). Whole blood samples were stained and incubated for 15 min, then lysed and fixed. Data were collected with a Navios cytometer (Beckman Coulter) and analysed with Kaluza software (Beckman Coulter).

### Follow-up

The main outcome measure was all-cause mortality at 18 months following admission for CAP. Mortality data were obtained by reviewing medical records or by telephone contact. Time to death was defined as the interval between the date of pneumonia diagnosis and the date of death. All patients who remained alive at 18 months were administratively censored. No patients were lost to follow-up.

### Statistical analysis

Continuous variables are expressed as means with standard deviations (SD). Categorical variables are expressed as percentages relative to the total sample. Continuous variables were compared using the Mann–Whitney U test. Categorical variables were compared with chi-square tests and Fisher’s exact test, when necessary.

Multivariate logistic regression analyses were performed to evaluate the association between the study variables and outcome measures. We were particularly interested in assessing the effect of IRP adjusted for potential cofounders given its clinical relevance as the main study objective. Variables that were statistically significant on the univariate analyses (*p* ≤ 0.05) were included in the multivariate analyses. For highly correlated variables, we retained only the variable with the greatest clinical relevance and/or strongest statistical significance to avoid multicollinearity. Several different multivariate logistic regression models were created using the enter method to identify the set of predictor variables that best explained the dependent variable.

Models were compared according to the statistical significance of the variables, deviance, absence of collinearity (VIF < 10), and the presence of potential cofounders. The final models were selected based on their overall goodness-of-fit and evaluated through calibration (Hosmer-Lemeshow test) and discrimination (area under the ROC curve).

The final multivariate logistic regression model predicting IRP included two variables: sex and lymphocyte count > 1500 × 109/L. The final multivariate logistic regression model predicting 18-month mortality included the following variables: creatinine clearance < 30 mL/min/1.72 m^2^; BI; serum albumin < 35 g/L; and IRP. Although COVID-19 status was initially included in the model, it was ultimately excluded due to lack of statistical significance.

Survival time was defined as the interval between the date of CAP diagnosis and the date of last contact or death. Adjusted survival curves were used to estimate cumulative survival. The final 18-month mortality model was evaluated using Cox proportional hazards regression, reporting hazard ratios (HR) with 95% confidence intervals (CI). Proportional hazards assumptions were verified through Schoenfeld residuals analysis. Model discrimination was assessed using Harrell’s C-statistic, and calibration through comparison of predicted vs. observed events across risk quintiles. Analyses were repeated in non-COVID-19 patients.

Statistical analyses were performed using SPSS Statistics, v. 27 (Armonk, NY, USA: IBM Corp) for adjusted survival curves, Cox proportional hazards modelling, and primary descriptive statistics. Calibration and discrimination metrics (Harrell’s C-static, Somers’ D) were performed using STATA, v. 17 (College Station, TX, USA: STATA Corp). P values ≤ 0.05 were considered significant.

## Results

A total of 272 patients were eligible for inclusion at hospital admission. Of these, 14 patients were excluded due to death during hospitalisation. Following a second check of the exclusion criteria, an additional 11 patients were excluded. Therefore, 247 patients were invited to participate and 175 accepted. Two patients died before evaluation, and 30 patients did not present at the scheduled evaluation. A total of 143 patients (79 males; 55.2%) completed the evaluation and were followed for 18 months. The mean (SD) age was 77.6 (7.9) years. The main clinical and laboratory characteristics of the cohort are shown in Tables 1 and 2. The specific microbiological aetiology was identified in 62 patients, as follows: SARS-CoV-2 (*n* = 41, 28.5%); Streptococcus pneumoniae (*n* = 10, 6.8%); Legionella pneumoniae (*n* = 7, 4.7%); and other (*n* = 4, 2.7%). The differences between patients with SARS-CoV-2 vs. other aetiologies are shown in Supplementary Table 1.

**Table 1:** Main clinical characteristics of the full cohort stratified by IRP status

Baseline characteristics	All participants (*N* = 143)	IRP+ (*n* = 67)	IRP- (*n* = 76)	*P*
Age, mean years (SD)	77.6 (7.9)	78.5 (7.5)	76.4 (7.5)	0.324
Female sex, n (%)	64 (44.8)	39 (58.2)	25 (32.9)	0.003
Influenza vaccine, n (%)	91 (63.6)	40 (59.7)	51 (67.1)	0.388
13v Pneumococcal vaccine, n (%)	8 (5.6)	2 (3)	6 (7.9)	0.283
23v Pneumococcal vaccine, n (%)	82 (57.3)	40 (59.7)	42 (55.3)	0.615
Charlson comorbidity index, mean (SD)	1.73 (1.82)	1.66 (1.6)	1.71 (2.0)	0.988
Main comorbidities:				
Acute myocardial infarction, n (%)	13 (9.1)	5 (7.5)	8 (10.5)	0.525
Congestive heart failure, n (%)	23 (16.1)	8 (11.9)	15 (19.7)	0.257
Peripheral vascular disease, n (%)	10 (7.0)	1 (1.5)	9 (11.8)	0.02
Cerebrovascular disease, n (%)	14 (9.8)	3 (4.5)	11 (14.5)	0.052
Dementia, n (%)	8 (5.6)	5 (7.5)	3 (3.9)	0.474
COPD, n (%)	41 (28.7)	22 (32.8)	19 (25)	0.356
Rheumatic disease, n (%)	4 (2.8)	2 (3)	2 (2.6)	1
Peptic ulcer, n (%)	3 (2.1)	1 (1.5)	2 (2.6)	1
Mild to moderate diabetes, n (%)	33 (23.1)	19 (28.4)	14 (18.4)	0.170
Mild hepatopathy, n (%)	2 (1.4)	2 (3)	0	0.218
Chronic kidney disease^a^, n (%)	17 (11.9)	8 (11.9)	9 (11.8)	1
Diabetes with chronic complications, n (%)	10 (7)	6 (9)	4 (5.3)	0.516
Tumour	9 (6.3)	3 (4.5)	6 (7.9)	0.502
Leukaemia, n (%)	2 (1.4)	1 (1.5)	1 (1.3)	1
PSI, mean (SD)	97.9 (25.9)	100.8 (28.3)	95.4 (23.6)	0.271
SARS-CoV2 positivity, n (%)	41 (28.7)	16 (23.8)	25 (32.8)	0.269
Barthel Index, mean (SD)	83.7 (22.8)	81.6 (24.6)	86.5 (20.0)	0.470
Institutionalisation^b^, n (%)	28 (19.6)	15 (22.3)	13 (17.1)	0.527
Frailty^c^, n (%)	51 (35.7)	25 (37.3)	26 (34.2)	0.729
BMI, mean (SD)	26.94 (5.27)	27.4 (5.6)	26.6 (4.77)	0.780
MNA, mean (SD)	20.49 (5.09)	20.3 (5.2)	20.8 (4.8)	0.372

*Abbreviations*: *SD* Standard deviation, *COPD* Chronic obstructive pulmonary disease, *PSI* Pneumonia Severity Index, *BMI* Body mass index, *MNA* Mini Nutritional Assessment

^a^Chronic kidney disease assessed according to CCI (level of serum creatinine > 265 pmol/L, on dialysis, kidney transplant recipient, or uraemia)

^b^ Institutionalization in either nursing home or long-term facilities

^c^ Frailty according to Fried’s criteria

**Table 2:** Main laboratory characteristics in the full cohort and according to IRP status

Laboratory characteristics:	Full cohort (*n* = 143)	IRP+ (*n* = 67)	IRP- (*n* = 76)	*P*
CRP mg/L, mean (SD)	11.61(22.6)	12.84 (28.6)	19.31 (14.8)	0.942
Creatinine pmol/L, mean (SD)	90.6 (43.7)	87.18 (39.4)	93.5 (49.1)	0.332
Clearance mL/min/1.72 m^2^, mean value (SD)	70.2(21.3)	69.8 (20.7)	70.7 (22.4)	0.627
Cholesterol mmol/L, mean (SD)	4.99 (1.2)	5.11 (1.2)	4.88 (1.1)	0.128
Triglycerides mmol/L, mean (SD)	1.46 (0.7)	1.61 (0.8)	1.32 (0.6)	0.026
Calcium mmol/L, mean (SD)	2.31 (0.12)	2.33 (0.12)	2.28(0.16)	0.045
Albumin g/L, mean (SD)	38.8 (3.9)	39.2 (3.9)	38.6 (3.9)	0.198
Pre-albumin g/L, mean (SD)	0.25(0.06)	0.25 (0.07)	0.25 (0.06)	0.730
Vitamin D nmol/L, mean value (SD)	50.5 (30.8)	47.9 (31.2)	53.5 (31.4)	0.362
Hb g/l, mean (SD)	124.6 (18.0)	122.4 (20.3)	126.9 (16.1)	0.210
Leucocytes cells/µL, mean (SD)	7250.5 (2659.9)	7529.5 (2435.5)	6803.5 (2712.9)	0.091
Lymphocytes cells/µL, mean (SD)	1614.0 (715.6)	1935.3 (745.2)	1351 (587.5)	**< **0.001
Lymphopenia, n (%)	66 (46.2)	20 (29.8)	46 (60.5)	**< **0.001
IRP criteria:				
Positive CMV serology, n (%)	124 (86.7)	67 (100)	57 (75)	**< **0.001
Lymphocytes T CD28-^a^, n (%)	68 (47.5)	65 (97.0)	3 (4.0)	**< **0.001
Lymphocytes T CD8 + ^b^, n (%)	27 (18.8)	26 (38.8)	1 (1.3)	**< **0.001
Lymphocytes T CD4/CD8 < 1, n (%)	22 (15.3)	20 (29.8)	2 (2.6)	**< **0.001

*Abbreviations*: *SD* Standard deviation, *CRP* C-reactive protein, *Hb *Haemoglobin, *IRP* Immune risk phenotype, *CMV *Cytomegalovirus

^a^TCD8 CD28- lymphocyte count > 300 / µl

^b^CD8 T-cells lymphocyte count > 600/ µl

Twenty-two (15.4%) patients had a CD4:CD8 ratio < 1. Sixty-seven patients (46.8%) met criteria for IRP. Compared to men, a higher proportion of women met criteria for IRP (60.9% vs. 35.4%, *p* = 0.003), CMV seropositivity (93.7% vs. 81.0%, *p* = 0.028), and T lymphocytes CD28- (62.5 vs. 35.8%, *p* = 0.002). No other sex-related differences in baseline characteristics were observed.

The main differences between patients presenting with and without IRP are shown in Tables 1 and 2.

On the multivariate analyses, lymphocyte count > 1500 × 10^9^/L (odds ratio [OR]: 3.40; 95% confidence interval [CI: 1.6–6.9, *p* < 0.001) and female sex (OR, 2.65; 95% CI: 1.3–5.3, *p* = 0.007) were identified as risk factors for IRP. The model showed acceptable discrimination (AUC-ROC, *p* = 0.70), and excellent calibration (Hosmer-Lemeshow, *p* = 0.99).

At 18 months post-diagnosis, 27 of the 143 patients had died (18.8%). Of these, 22 died in the first 12 months (15.4%). The causes of death were as follows: pneumonia (*n* = 11; 4 due to SARS-CoV2); other infections (*n* = 2); fractures (*n* = 2); cardiovascular disease (*n* = 1); other causes (*n* = 6); and unknown (*n* = 5).

Table 3 shows the characteristics and main differences between survivors and non-survivors. Malnutrition and risk of malnutrition (according to MNA) were significantly less common among survivors than non-survivors (10.3% vs. 66.6%, *p* < 0.001 and 50.9 vs. 77.7%, *p* < 0.001, respectively).

**Table 3:** Patient characteristics according to survival status at 18 months

Baseline characteristic	Survivors (*n* = 116)	Non-survivors (*n* = 27)	*P*
Age, mean years (SD)	76.32 (7.37)	82.4 (8.4)	**< **0.001
Female sex, n (%)	53 (45.7)	11 (40.7)	0.674
Influenza vaccine, n (%)	66 (56.9)	25 (92.6)	**< **0.001
13v Pneumococcal vaccine, n (%)	6 (5.2%)	2 (7.4)	0.646
23v Pneumococcal vaccine, n (%)	59 (50.9)	23 (85.2)	**< **0.001
Charlson comorbidity index, mean (SD)	1.35 (1.56)	3.16 (2.15)	**< **0.001
Main comorbidities:			
Acute myocardial infarction, n (%)	6 (5.2)	7 (25.9)	0.003
Congestive heart failure, n (%)	16 (13.8)	7 (25.9)	0.146
Peripheral vascular disease, n (%)	4 (3.4)	6 (22.2)	0.003
Cerebrovascular disease, n (%)	12 (10.3)	2 (7.4)	1
Dementia, n (%)	3 (2.6)	5 (18.5)	0.006
COPD, n (%)	32 (27.6)	9 (33.3)	0.637
Rheumatic disease, n (%)	3 (2.6)	1 (3.7)	0.571
Peptic ulcer, n (%)	1 (0.9)	2 (7.4)	0.091
Mild to moderate diabetes, n (%)	26 (22.4)	7 (25.9)	0.8
Mild hepatopathy, n (%)	2 (1.7)	0	1
Chronic kidney disease^a^, n (%)	7 (6)	10 (37)	**< **0.001
Diabetes with chronic complications, n (%)	4 (3.4)	6 (22.2)	0.003
Tumour	9 (7.8)	0	0.208
Leukaemia, n (%)	1 (0.9)	1 (3.7)	0.343
PSI, mean (SD)	93.2 (21.7)	118.1 (32.8)	**< **0.001
SARSCoV2 positivity, n (%)	41 (35.3)	0	**< **0.001
Frailty^b^, n (%)	32 (27.6)	19 (70.4)	**< **0.001
BMI, mean (SD)	27.73 (4.81)	23.97 (5.70)	**< **0.001
Barthel Index, mean (SD)	88.77 (16.10)	60.0 (29.54)	**< **0.001
MNA, mean (SD)	21.98 (3.91)	14.52 (4.78)	**< **0.001
Cholesterol mmol/L, mean (SD)	5.22 (1.07)	3.98 (1.27)	**< **0.001
Triglycerides mmol/L, mean (SD)	1.50 (0.79)	1.29 (0.59)	0.188
CRP mg/L, mean (SD)	6.73 (11.5)	29.7 (42.1)	**< **0.001
Calcium mmol/L, mean (SD)	2.32 (0.14)	2.21 (0.13)	**< **0.001
Albumin g/L, mean (SD)	39.94 (3.24)	34.7 (3.84)	**< **0.001
Pre-albumin g/L, mean (SD)	0.26 (0.06)	0.20 (0.06)	**< **0.001
Vitamin D nmol/l, mean (SD)	52.94 (32.0)	41.75 (26.7)	0.127
Hb, mean (SD)	126.83 (17.63)	115.68 (19.07)	0.001
Leucocytes, mean (SD)	6906.2 (2243.2)	8243.4 (3639.6)	0.004
Lymphocytes, mean (SD)	1688.0 (722.9)	1296.3 (596.9)	0.008
Lymphopenia, n (%)	48 (41.3)	18 (66.6)	0.018
IRP, n (%)	52 (44.8)	15 (55.5)	0.314
IRP criteria:			
Positive CMV serology, n (%)	100 (86.2)	24 (88.8)	0.712
Lymphocytes T CD28- ^c^, n (%)	54 (46.1)	14 (51.8)	0.647
Lymphocytes T CD8 + ^d^, n (%)	23 (19.8)	4 (14.8)	0.549
Lymphocytes T CD4/CD8 < 1, n (%)	17 (14.6)	5 (18.5)	0.616

*Abbreviations*: *SD* Standard deviation, *COPD* Chronic obstructive pulmonary disease, *PSI* Pneumonia Severity Index, *BMI* Body mass index, *MNA* Mini Nutritional Assessment, *CRP* C-reactive protein, *Hb* Haemoglobin, *IRP* immune risk phenotype, *CMV *Cytomegalovirus

^a^Chronic kidney disease assessed according to CCI (level of serum creatinine > 265 pmol/L, being on dialysis, being a recipient of renal transplantation, or presenting uraemia)

^b^ Frailty definition according to Fried’s criteria

^c^TCD8 CD28- lymphocytes count > 300 / µl

^d^ CD8 T-cells lymphocyte count > 600/ µl

On the multivariate logistic regression analysis for 18-month mortality, after testing different combinations, the best-fitting model (AUC-ROC, *p* = 0.78; Hosmer-Lemeshow, *p* = 0.34) included the following variables: creatinine clearance < 30 mL/min1.72m^2^ (OR, 16.26; 95% CI: 2.84-93.0; *p* = 0.002), BI < 60 points (OR, 3.58; 95% CI: 1.06–12.09; *p* = 0.04), serum albumin < 35 g/L (OR, 4.60; 95% CI: 1.21–17.43; *p* = 0.025), and IRP (OR 2.41; 95% CI: 0.85–6.86; *p* = 0.09).

**Fig. 1:**
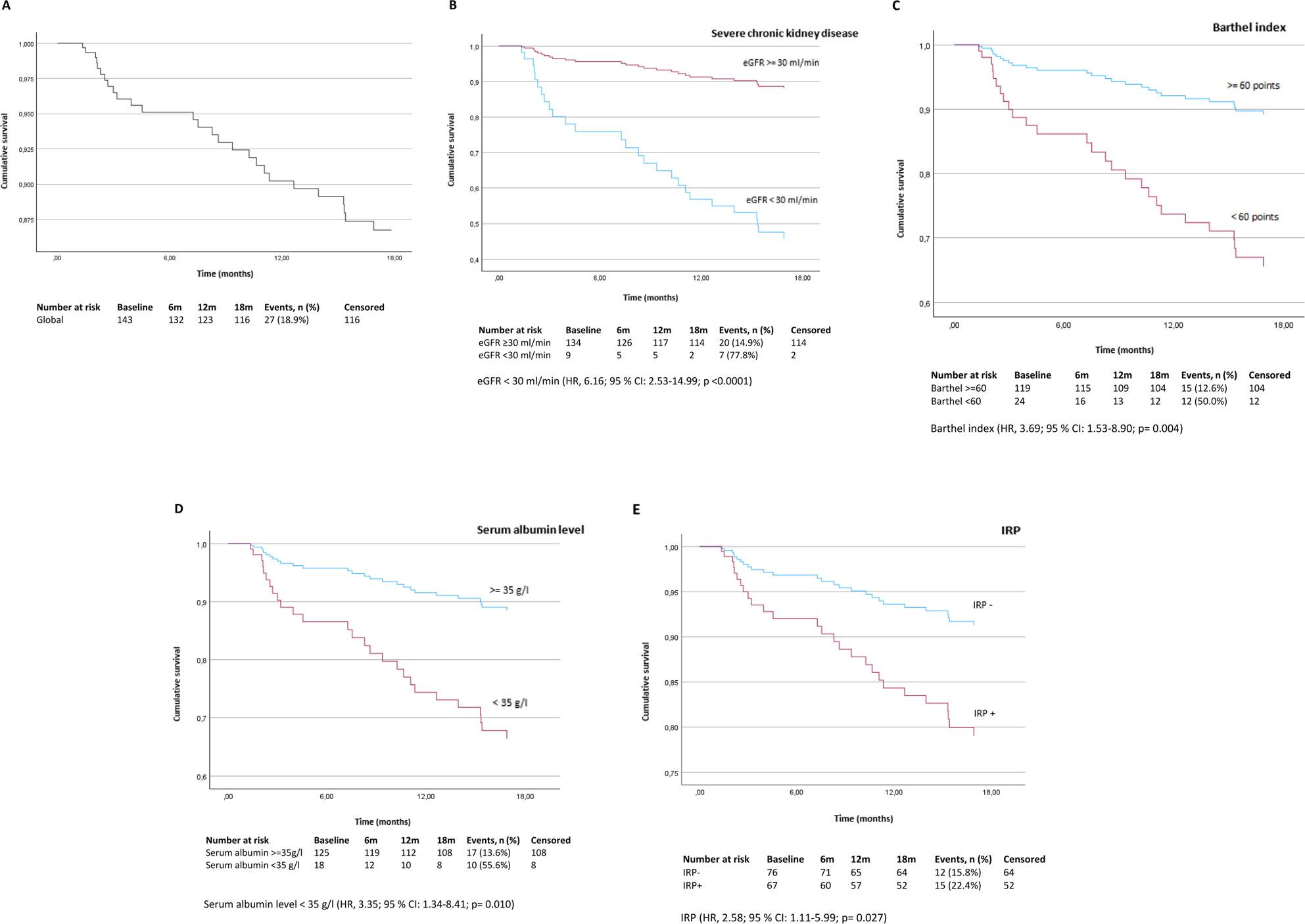
Adjusted survival curves for 18-month survival. **A **General survival curve. **B **Survival curves stratified by presence of severe chronic kidney disease (< eGFR 30 mL/min vs. ≥ 30 mL/min). **C** Survival curves stratified by Barthel Index < 60 points vs. ≥ 60 points. **D **Survival curves stratified by serum albumin level < 35 g/L vs. ≥ 35.1 g/L. **E **Survival curves stratified by positive IRP status

Figure 1 shows adjusted survival curves for 18-month survival, including the general survival curve for the cohort (A) and adjusted survival curves stratified by severe chronic kidney disease *(eGFR < 30 ml/ min 1.72 m*^*2*^*)* (B), Barthel index score *(< 60 points)* (C), hypoalbuminemia *(< 35 g/L)* (D), and IRP (E).

On the Cox proportional hazards model, the following variables were independent predictors of 18-month mortality: creatinine clearance < 30 mL/min 1.72 m^2^ (hazard ratio [HR], 6.16; 95% CI: 2.53–14.99; p = < 0.001); BI < 60 points (HR, 3.69; 95% CI: 1.53–8.90; *p* = 0.004); low serum albumin (HR, 3.35, 95% CI: 1.34–8.41; *p* = 0.010), and IRP (HR, 2.58; 95% CI: 1.11–5.99; *p* = 0.027).

The Cox model showed good overall fit (HR, 2.71; 95% CI: 1.99–3.71), with acceptable calibration on average (slope, 1.0; 95% CI: 0.69–1.31). Graphical assessment revealed some overestimation of survival in high-risk patients, and discrimination remained strong (Harrell’s C, 0.76; Somers’ D, 0.52).

Sensitivity analyses excluding patients with COVID-19-related CAP showed a broadly similar pattern of associations on the Cox proportional hazards model, although the Barthel Index and IRP were no longer statistically significant. Detailed results are provided in Supplementary Table 2.

Additional Cox sensitivity analyses, including models adjusted for age and sex, as well as alternative models incorporating frailty status and BMI are provided in Supplementary Table 3.

Regarding the IRP construct, no significant associations were observed between the individual immune components of the IRP and 18-month mortality (Supplementary Table 4). Likewise, none of the individual IRP components contributed significantly when individually incorporated into the Cox regression models (Supplementary Table 5).

## Discussion

This study provides a detailed description of long-term mortality outcomes in a cohort of older adults following hospital discharge for CAP. To our knowledge, this is the first study to report 12-and 18-month mortality outcomes in this population after the epidemiological shift caused by the COVID-19 pandemic. Our findings show that the presence of IRP, together with low serum albumin levels, poor functional status, and severe kidney disease, are key prognostic factors, underscoring the clinical relevance of these factors in geriatric populations.

Long-term mortality rates in this cohort were 15.3% at 12 months and 18.8% at 18 months, consistent with the mortality rates reported in similar studies (12%-17% and 17.3%, respectively) [3–7, 24, 25]. However, the main cause of death in nearly half of patients was infectious disease, mainly recurrent pneumonia; by contrast, fractures and cardiovascular disease accounted for only 7.4% and 3.7% of deaths, respectively. The fact that infectious diseases were the leading cause of death in our study is noteworthy. Although Adamuz et al. reported similar findings (48% of deaths attributable to infectious disease) [26], our findings stand in contrast with many earlier studies in which cardiovascular disease and chronic obstructive pulmonary disease (COPD) were the leading causes of death [3, 6, 7, 27, 28]. Several factors could explain these differences. First, most of the studies that have assessed long-term mortality after CAP were published more than a decade ago and recent advances in the management of chronic conditions—particularly cardiovascular disease—may have reduced post-CAP mortality rates. Second, we included only older adults (mean age, 77.6 years) who required hospitalization, unlike prior studies that included broader age ranges and/or outpatient populations. Finally, the epidemiological context (i.e., during the COVID-19 pandemic) in place during the study period may have influenced mortality outcomes. Although only 15% of long-term deaths in our sample were directly attributable to COVID-19, pandemic-related social isolation may have compromised nutritional status, physical activity, and mood, thereby impairing immunity [29, 30], which would at least partially explain the higher burden of infectious diseases.

Several long-term risk factors for mortality following an episode of CAP have been identified, including increasing age and comorbidities, both of which are well-established predictors of worse outcomes [3, 24, 31]. Interestingly, age was not an independent predictor of mortality in our model. This finding has already been reported in other CAP studies [3, 7, 9, 26, 32, 33], suggesting that age “per se” is not an independent risk factor in this population [3]. In this regard, it is worth noting that age is not included in the Walter Index (WI) [34], a widely used prognostic tool for 12-month mortality in hospitalized adults. Instead, the WI includes the three risk factors identified in our model: severe kidney disease, poor functional status, and hypoalbuminemia. Many other studies have also found that these same three risk factors are predictors of long-term mortality in older patients hospitalized for pneumonia [4, 7, 9, 26, 27, 32, 35]. These variables appear to interact in a self-perpetuating cycle that sustains chronic inflammation, drives functional decline (often accompanied by dysphagia), and promotes malnutrition, ultimately accelerating immune aging [36].

The predominance of recurrent infections as the primary cause of long-term mortality in a large proportion of our sample, together with the presence of the associated risk factors, suggests that immunosenescence plays a key role in this process. Pneumonia may be a clinical indicator of underlying immune vulnerability, thus underscoring the need to better understand immunosenescence and to identify reliable biomarkers. That said, identifying a robust biomarker is highly challenging due to the heterogeneity of immune aging and the lack of a single parameter that fully captures its complexity.

The concept of an immune risk phenotype was originally developed as a practical tool to assess predictive blood markers of immunological aging [37, 38]. IRP is characterized by an inverted CD4:CD8 ratio and the accumulation of terminally differentiated resistant CD8 + CD28- cells, probably driven by persistent CMV infection [12, 38]. In the study by Plonquet et al. [11], one out of every four patients admitted to a geriatric rehabilitation unit after transfer from an acute medical or orthopaedic surgical unit met criteria for IRP, conferring a higher risk of nosocomial respiratory infections. Moreover, the prevalence of IRP was even higher in patients who went on to develop a respiratory nosocomial infection (38% vs. 25%) [11]. In our study, which was comprised of older adults recently hospitalized for CAP, nearly half of the patients met criteria for IRP. The high prevalence of CMV seropositivity observed in our cohort may have reduced its discriminative contribution within the IRP definition, suggesting that the prognostic signal captured by IRP was more strongly influenced by T-cell remodelling markers. In addition, female sex and higher lymphocyte counts were associated with IRP in our cohort, findings that may reflect an expansion of dysfunctional lymphocyte populations, which are consistent with the higher seropositivity observed among women [10, 39]. Although CD28- T-cell expansion and CD4/CD8 imbalance showed the strongest tendency toward association with mortality, none of the individual immune components reproduced the prognostic signal observed for the composite IRP construct. These findings support the interpretation that IRP may reflect the combined effect of several interacting immune alterations rather than a single isolated parameter, in line with current concepts describing immunosenescence as a heterogeneous process [40].

This study has several limitations. First, it was a single centre study (tertiary hospital), which may limit its generalizability to other settings. Second, the emergence of the COVID-19 pandemic during the recruitment period posed significant challenges, including the dilemma of whether to include patients with COVID-19-related CAP, given that our inclusion criteria did not specify the CAP aetiology. Ultimately, we decided to include those patients while carefully documenting the aetiology (SARS-CoV-2). While this decision provided valuable real-world data, it resulted in a more heterogeneous study sample. Recruitment and follow-up during the pandemic were difficult due to pandemic-related restrictions, and patients with COVID-19 CAP, particularly in the early waves, were younger and fitter. Notably, at 18-months, no deaths were observed in the COVID-19 patients and this aetiology was not a predictor of long-term mortality. These findings are consistent with previous studies [32, 41–43], suggesting that COVID-19 and CAP have similar or even lower long-term mortality rates in older adults who survive the critical first 30 days. To further address the potential heterogeneity introduced by inclusion of COVID-19 related CAP, sensitivity analyses excluding these patients from the Cox model for 18-month mortality were performed, showing a broadly similar pattern of associations. However, some of the predictors no longer reached statistical significance, likely due to a decrease statistical precision rather than to a substantial change in the underlying pattern of associations.

A third limitation was the relatively limited number of mortality events, which may have affected the precision of some estimates in the multivariable analyses. Final models were therefore restricted to a limited number of clinically relevant and non-collinear variables to reduce redundancy and minimize overfitting. Although the prognostic effect observed for IRP was moderate, the time-to-event Cox approach appeared more informative in this setting because it provides data on the longitudinal evolution of mortality during follow-up.

Notwithstanding these limitations, this study also has several strengths, including the prospective study design, the integration of geriatric assessment with immune phenotyping, the comprehensive nutritional assessment, the acceptable performance models, and a complete follow-up of all patients. Considered together, we believe that the rigorous study design provides a strong framework to help better understand long-term outcomes and the multidimensional vulnerability of older adults hospitalised for CAP.

## Conclusion

The findings of this study highlight the growing importance of infectious diseases as a cause of long-term mortality in older adults after hospitalisation for CAP, especially as advances in chronic disease management reshape the epidemiological landscape. The predictive model developed in this study further reinforces the relevant role of established factors—including functional status, kidney function, and serum albumin levels—in determining mortality outcomes, while also providing data to suggest that IRP-related immune aging signatures may contribute to long-term vulnerability after CAP.

These findings suggest that routine assessment of IRP status could potentially improve risk stratification and open new avenues for intervention and research to improve clinical outcomes in this vulnerable population. 

## Supplementary Information

Supplementary Material 1.

## Data Availability

The datasets used and/or analysed during the current study are available from the corresponding author upon reasonable request.

## Abbreviations

CAP: Community acquired pneumonia
CMV: Cytomegalovirus
IRP: Immune Risk Phenotype
HIV: Human Immunodeficiency Virus
BI: Barthel Index
PSI: Pneumonia Severity Index
COVID-19: Coronavirus Disease 2019
CGA: Comprehensive Geriatric Assessment
CGN: General Nutritional Assessment
CCI: Charlson Comorbidity Index
CKD: Chronic Kidney Disease
GFR: Glomerular Filtration Rate
BMI: Body Mass Index
MNA: Mini Nutritional Assessment
SD: Standard Deviation
CI: Confidence Interval
COPD: Chronic Obstructive Pulmonary Disease
WI: Walter Index
QoL: Quality of Life
