# Supplementary material for: Immune risk phenotype and long-term mortality after community-acquired pneumonia in older adults: clinical and immune determinants
Source: Immun Ageing. 2026 Jun 2;23:27. doi: 10.1186/s12979-026-00574-8 (PMC13463403; doi:10.1186/s12979-026-00574-8)
Supplement: Supplementary file 1 [file 12979_2026_574_MOESM1_ESM.docx]

**SUPPLEMENTARY DATA**

**Supplementary Table 1: Differences between patients with and without SARS-CoV-2**

| Baseline characteristics | SARS-CoV-2 +  (n=41) | Non-SARS-CoV-2 -  (n=102) | p |
| --- | --- | --- | --- |
| Age, mean years (SD) | 73.3 (5.2) | 78.6 (8.3) | **<0.001** |
| Female sex, n (%) | 18 (43.9) | 46 (45.0) | 0.897 |
| Smoking status, n (%) | 0 | 7 (6.9) | 0.088 |
| Previous institutionalization, n (%) | 1 (2.4) | 12 (11.7) | 0.111 |
| Charlson Comorbidity Index, mean (SD) | 0.98 (1.23) | 2.01 (1.9) | **0.002** |
| PSI, mean (SD) | 84.5 (17.4) | 103.5 (26.8) | **<0.001** |
| Barthel Index at 2 months, mean (SD) | 96.7 (10.4) | 91.6(19.0) | 0.112 |
| Institutionalization at 2 months, n (%) | 1 (2.4) | 27 (26.4) | **<0.001** |
| BMI, mean (SD) | 29.0 (5.0) | 26.17 (5.1) | **0.003** |
| MNA, mean (SD) | 23.9 (2.4) | 19.2 (5.1) | **<0.001** |
| CRP mg/L, mean (SD) | 6.25 (14.5) | 13.7 (24.9) | **<0.001** |
| Creatinine pmol/L, mean (SD) | 80.24 (26.0) | 94.8 (48.6) | 0.210 |
| Clearance ml/min/1.72 m^2^, mean (SD) | 76.5 (17.1) | 67.7 (22.4) | 0.041 |
| Cholesterol mmol/L, mean (SD) | 5.37 (0.9) | 4.84 (1.2) | **0.007** |
| Triglycerides mmol/L, mean (SD) | 1.45 (0.8) | 1.46 (0.7) | 0.860 |
| Calcium mmol/L, mean (SD) | 2.28 (0.11) | 2.32 (0.13) | 0.06 |
| Albumin g/L, mean (SD) | 39.99 (3.4) | 38.44 (4.1) | **0.013** |
| Pre-albumin g/L, mean (SD) | 0.27 (0.06) | 0.24 (0.07) | **0.033** |
| Vitamin D nmol/L, mean (SD) | 43.48 (19.6) | 55.18 (34.6) | 0.258 |
| Hb, mean (SD) | 128.7 (13.2) | 132.0 (19.4) | 0.066 |
| Leucocytes, mean (SD) | 6759.7 (1952.4) | 7447.7 (2881.0) | 0.119 |
| Lymphocytes, mean (SD) | 1672.4 (671.0) | 1590.6 (734.6) | 0.389 |
| Lymphopenia, n (%) | 15 (36.6) | 51 (50.0) | 0.194 |
| IRP, n (%) | 16 (39.0) | 51 (50.0) | 0.269 |
| IRP criteria: |  |  |  |
| Positive CMV serology, n (%) | 39 (95.1) | 85 (83.3) | 0.099 |
| Lymphocytes T CD28-^c^, n (%) | 17 (41.4) | 51 (50.4) | 0.359 |
| Lymphocytes T CD8+^d^, n (%) | 6 (14.6) | 21 (20.5) | 0.485 |
| Lymphocytes T CD4/CD8 <1, n (%) | 7 (17.0) | 15 (14.7) | 0.451 |

Abbreviations: SD, standard deviation; COPD, chronic obstructive pulmonary disease; PSI, Pneumonia Severity Index; BMI, body mass index; MNA, Mini Nutritional Assessment; CRP, C-reactive protein; Hb, haemoglobin; IRP, immune risk phenotype; CMV, cytomegalovirus.

^a^Chronic kidney disease assessed according to CCI (level of serum creatinine > 265 pmol/L, being on dialysis, being a recipient of renal transplantation, or presenting uraemia). ^b^ Frailty definition according to Fried’s criteria ^c^TCD8 CD28- lymphocytes count > 300 / µl. ^d^ CD8 T-cells lymphocyte count > 600/ µl

**Supplementary Table 2: Comparisons of Cox proportional hazard models in the full cohort and in non-COVID patients**

|  | **Full cohort (n=143)** | | | **Non-COVID patients (n=102)** | | |
| --- | --- | --- | --- | --- | --- | --- |
| **Variable** | **HR** | **95% Confidence Interval** | **p** | **HR** | **95% Confidence Interval** | **p** |
| Creatinine clearance < 30 mL/min 1.72 m^2^ | 6.12 | 2.53-14.9 | **<0.001** | 6.28 | 2.56-15,4 | **0.000** |
| Barthel Index < 60 points | 3.69 | 1.53-8.89 | **0.004** | 2.05 | 0.76-5.47 | 0.152 |
| Low serum albumin | 3.35 | 1.33-8.41 | **0.010** | 3.87 | 1.47-10.21 | **0.006** |
| IRP | 2.58 | 1.14-5.99 | **0.02** | 1.93 | 0.82-4.53 | 0.128 |

**Supplementary Table 3: Additional Cox proportional Hazard Models for 18-month mortality**

|  | HR | 95% Confidence Interval | p |
| --- | --- | --- | --- |
| Model A: including sex and age |  |  |  |
| Creatinine Clearance < 30 mL/min/1.72 m^2^ | 5.86 | 2.42-14.20 | **<0.001** |
| Barthel Index < 60 points | 2.96 | 1.08-8.11 | **0.035** |
| Low serum albumin | 3.15 | 1.23-8.01 | **0.016** |
| IRP | 2.90 | 1.13-7.40 | **0.026** |
| Sex | 1.71 | 0.71-4.14 | 0.230 |
| Age | 1.04 | 0.98-1.10 | 0.163 |
| Model B: including Frailty instead of Barthel Index |  |  |  |
| Creatinine Clearance < 30 mL/min/1.72 m^2^ | 3.80 | 1.49-9.43 | **0.005** |
| Frailty | 3.22 | 1.33-7.80 | **0.009** |
| Low serum albumin | 4.46 | 1.86-10.73 | **<0.001** |
| IRP | 2.25 | 0.97-5.23 | **0.058** |
| Model C: including BMI instead of Albumin |  |  |  |
| Creatinine Clearance < 30 mL/min/1.72 m^2^ | 6.41 | 2.57-16.01 | **0.000** |
| Barthel Index < 60 points | 5.29 | 2.26-12.35 | **0.000** |
| BMI | 0.86 | 0.79-0.93 | **0.000** |
| IRP | 2.43 | 1.05-5.64 | **0.039** |
|  |  |  |  |
|  |  |  |  |
|  |  |  |  |

**Supplementary Table 4: Correlations between the individual immune components of the IRP and 18-month mortality**

| Immune components of the IRP | Spearman’s rho | P |
| --- | --- | --- |
| CMV serology | -0.076 | 0.450 |
| Lymphocytes T CD28- | 0.068 | 0.5 |
| Lymphocytes T CD8+ | 0.137 | 0.170 |
| Ratio of Lymphocytes T CD4/CD8 | 0.03 | 0.762 |
|  |  |  |

**Supplementary Table 5: Additional Cox proportional Hazard models including the individual components of the IRP construct**

| Cox proportional hazard models | HR | 95% Confidence Interval | p |
| --- | --- | --- | --- |
| Model A: CMV serology |  |  |  |
| Positive CMV serology | 1.73 | 0.50-6.02 | 0.390 |
| Barthel Index < 60 points | 3.25 | 1.40-7.53 | 0.006 |
| Low serum albumin | 2.81 | 1.17-6.74 | **0.021** |
| Creatinine Clearance < 30 mL/min/1.72 m^2^ | 5.51 | 2.30-13.24 | **<0.001** |
| Model B: Lymphocytes T CD28- |  |  |  |
| Lymphocytes T CD28- ^a^ | 2.11 | 0.91-4.56 | 0.081 |
| Barthel Index < 60 points | 3.59 | 1.49-8.64 | **0.004** |
| Low serum albumin | 3.17 | 1.26-7.96 | **0.014** |
| Creatinine Clearance < 30 mL/min/1.72 m^2^ | 5.96 | 2.45-14.49 | **0.000** |
| Model C: Lymphocytes T CD8+ |  |  |  |
| Lymphocytes T CD8+ ^b^ | 0.88 | 0.29-2.65 | 0.822 |
| Barthel Index < 60 points | 3.13 | 1.31-7.45 | **0.010** |
| Low serum albumin | 2.55 | 1.03-6.31 | **0.042** |
| Creatinine Clearance < 30 mL/min/1.72 m^2^ | 5.40 | 2.23-1.07 | **0.000** |
| Model D: Ratio of Lymphocytes T CD4/CD8 |  |  |  |
| Ratio of Lymphocytes T CD4/CD8 < 1 | 2.78 | 0.93-8.34 | 0.067 |
| Barthel Index < 60 points | 3.98 | 1.58-1.04 | **0.003** |
| Low serum albumin | 2.52 | 1.10-6.05 | **0.038** |
| Creatinine Clearance < 30 mL/min/1.72 m^2^ | 6.22 | 2.49-15.54 | **0.000** |

**^a^** Lymphocytes T CD28**-** count > 300 / µl ; ^b^ Lymphocytes T CD8+ count > 600/ µl.
